# Microbial functional diversity: From concepts to applications

**DOI:** 10.1002/ece3.5670

**Published:** 2019-10-02

**Authors:** Arthur Escalas, Lauren Hale, James W. Voordeckers, Yunfeng Yang, Mary K. Firestone, Lisa Alvarez‐Cohen, Jizhong Zhou

**Affiliations:** ^1^ MARBEC CNRS Ifremer IRD University of Montpellier Montpellier Cedex 5 France; ^2^ Institute for Environmental Genomics and Department of Microbiology and Plant Biology University of Oklahoma Norman OK USA; ^3^ Water Management Research Unit SJVASC USDA‐ARS Parlier CA USA; ^4^ State Key Joint Laboratory of Environment Simulation and Pollution Control School of Environment Tsinghua University Beijing China; ^5^ Department of Environmental Science, Policy, and Management University of California Berkeley CA USA; ^6^ Department of Civil and Environmental Engineering University of California Berkeley CA USA; ^7^ Earth and Environmental Sciences Lawrence Berkeley National Laboratory Berkeley CA USA

**Keywords:** functional diversity, functional traits, microbial communities, theoretical frameworks of diversity, trait‐based ecology

## Abstract

Functional diversity is increasingly recognized by microbial ecologists as the essential link between biodiversity patterns and ecosystem functioning, determining the trophic relationships and interactions between microorganisms, their participation in biogeochemical cycles, and their responses to environmental changes. Consequently, its definition and quantification have practical and theoretical implications. In this opinion paper, we present a synthesis on the concept of microbial functional diversity from its definition to its application. Initially, we revisit to the original definition of functional diversity, highlighting two fundamental aspects, the ecological unit under study and the functional traits used to characterize it. Then, we discuss how the particularities of the microbial world disallow the direct application of the concepts and tools developed for macroorganisms. Next, we provide a synthesis of the literature on the types of ecological units and functional traits available in microbial functional ecology. We also provide a list of more than 400 traits covering a wide array of environmentally relevant functions. Lastly, we provide examples of the use of functional diversity in microbial systems based on the different units and traits discussed herein. It is our hope that this paper will stimulate discussions and help the growing field of microbial functional ecology to realize a potential that thus far has only been attained in macrobial ecology.

## Graphical abstract

Functional diversity is increasingly recognized in microbial ecology as the essential link between biodiversity patterns and ecosystem functioning. However, this concept has no clear definition in microbial systems which impairs its applicability. Here, we provide a synthesis on the concept of microbial functional diversity, from its origin to its application.

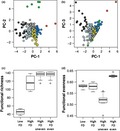

## 1 INTRODUCTION

Microbial communities play key roles in nearly every biogeochemical process that makes Earth inhabitable (Falkowski, Fenchel, & Delong, 2008). They mediate vital ecosystem processes such as primary production, decomposition, nutrient cycling, climate regulation, carbon storage, disease propagation, and pollutant transformation (Ducklow, 2008; Giller et al., 2004). Atop of that, microbes inhabiting the body of multicellular organisms are essential for the well‐being and survival of their hosts (Koskella, Hall, & Metcalf, 2017; McFall‐Ngai, 2015). Microbes exert major influences on ecological processes across space and time, owing to the fact that they represent the richest collection of chemical and molecular diversity in nature and their ability to interact and maintain dynamic relationships among themselves and with higher organisms.

The diversity of functions performed by organisms within ecosystems, coined as functional diversity, has been recognized as the missing link between biodiversity patterns and ecosystem functions (Bardgett & Van Der Putten, 2014; Lamarque, Lavorel, Mouchet, & Quetier, 2014; Loreau et al., 2001; Mouillot, Villéger, Scherer‐Lorenzen, & Mason, 2011) and is increasingly recognized as a core driver of ecosystem services (Carrara et al., 2015; Chapin et al., 2000; Díaz, Fargione, Chapin, & Tilman, 2006). There is an increasing recognition that patterns of functional diversity may provide a more powerful test of theory than taxonomic richness (Lamanna et al., 2014; Louca et al., 2018). However, despite many recommendations for more functionally oriented studies from leading microbial ecologists (Barberán et al., 2014; Boon et al., 2014; Burke, Steinberg, Rusch, Kjelleberg, & Thomas, 2011; Dinsdale et al., 2008; Fierer, Barberán, & Laughlin, 2014; Green, Bohannan, & Whitaker, 2008; Krause et al., 2014), microbial functional ecology still lags behind its macrobial counterpart. This is surprising considering it has been over 35 years since the first publication of a functional diversity index for microbes in 1981 (Troussellier & Legendre, 1981). Furthermore, it appears today ironical that before the molecular revolution traditional microbiologists identified taxa based on functional traits and phenotypic characteristics (Buchanan & Gibbons, 1975), while presently we attempt to infer the functional characteristics of taxa from their genomes and phylogeny (Aßhauer, Wemheuer, Daniel, & Meinicke, 2015; Langille et al., 2013). As a consequence, microbial functional ecology has not yet developed into a mature research field based on solid and consistent concepts which is notably due to inconsistencies in the way microbial functional diversity is defined and estimated (Bodelier, 2011; Krause et al., 2014).

In this opinion paper, we discuss the concept of microbial functional diversity, from its definition to its application, to generate ecological insights and better understand the functioning of microbial systems. Initially, we revisit to the original definition of functional diversity, highlighting two fundamental aspects, the ecological unit under study and the functional traits used to characterize it. Then, we discuss how the particularities of the microbial world disallow the direct application of the concepts and tools developed for macroorganisms. Next, we provide a synthesis of the literature on the types of ecological units and functional traits available in microbial functional ecology. Lastly, we provide examples of the use of functional diversity in microbial systems based on the different units and traits discussed herein.

## 2 CHALLENGES IN CHARACTERIZING MICROBIAL FUNCTIONAL DIVERSITY

### 2.1 General concept of functional diversity

Functional approaches for estimating biodiversity are based on the general premise that to understand the linkage between biodiversity and ecosystems functioning, the functions realized by organisms in natural systems are of greater interest than their identity. The term “functional diversity” has been widely used but most studies simply relied on presumed intuitive understanding of the term's meaning and thus there is no uniform definition, particularly in microbial ecology (Table S1, Petchey & Gaston, 2006). Carmona, de Bello, Mason, and Lepš (2016) provided a simple and operational definition of functional diversity as the “variation of traits between organisms,” which is “estimated as the variation of *traits* in the functional space occupied by an *ecological unit*.” Here, rather ambiguous notions appear of crucial importance for defining microbial functional diversity, ecological unit, and functional trait.

An ecological unit corresponds to any scale at which it is meaningful to estimate functional diversity, such as individual organisms, populations, species (or OTUs), communities, meta‐communities, geographical regions, and continents (Carmona et al., 2016). For macroorganisms, the ecological unit of choice is often the community and its functional diversity can be estimated considering the range, distribution, and variation of the traits carried by the species it contains, or the average trait values across species (i.e., community‐aggregated traits). Whatever the chosen ecological unit, it is now generally agreed that conceptualizing, defining, measuring, and ultimately understanding functional diversity depend on the measurement of functional traits (Mlambo, 2014; Petchey & Gaston, 2006), and the term “functional ecology” tends to be replaced by the more precise term “trait‐based ecology” (Shipley et al., 2016). The commonly used definition of functional traits describes those that “impact fitness of an organism via its effect on growth, reproduction, or survival” (Violle et al., 2007). This definition and its more recent variations (Carmona et al., 2016; Violle et al., 2017) state that functional traits should be measurable at the individual level, which is rarely an option for microorganisms. While quantitative traits (e.g., leaf area, morphological characteristics) are measured at the individual level and then averaged to estimate the trait value for the species, qualitative traits (e.g., phenological or behavioral) are more often estimated at the species level. This approach produces taxa‐traits matrices, that depict the functional attributes of taxa, which are then combined with taxa‐site matrices representing communities composition in order to estimate the functional diversity of communities using ad hoc indices (Mouchet, Villéger, Mason, & Mouillot, 2010; Villéger, Mason, & Mouillot, 2008).

In summary, functional approaches use traits to describe the role of ecological units in the functioning of natural systems. In the following sections, we will see why the definitions of functional diversity and functional traits currently used in macrobial ecology do not fit with the particularities of the microbial world and which aspects should be taken into consideration in order to improve our ability to characterize microbial functional diversity.

### 2.2 Toward a trait‐based approach of microbial functional diversity

Biodiversity is generally seen as a triad composed of taxonomic, phylogenetic, and functional diversity. Taxonomic and phylogenetic microbial diversities are both estimated using a single, highly conserved, marker genes (e.g., 16S rRNA for bacteria and archaea, ITS for fungi, and 18S rRNA for microbial eukaryotes; Findley et al., 2013). However, the wide range of traits and ecological strategies observed in microbes cannot be summarized by a single gene, as the depth of phylogenetic conservation varies across microbial traits (Goberna & Verdú, 2016) and depends on their complexity (Martiny, Jones, Lennon, & Martiny, 2015). For instance, simple traits that involve few functional genes tend to occur at a shallow depth in phylogenetic trees and are often not shared by all members of a given taxon (Martiny et al., 2015). For instance, the ability to produce alkaline phosphatase is encoded by a single gene (Torriani‐Gorini, Yagil, & Silver, 1994). Furthermore, as suggested by Young (2016), simple traits are more likely to be carried on phages, plasmids, or transposons, further facilitating modification of microbial genomes at or below the species level through horizontal gene transfer or HGT (Mourkas et al., 2019; Polz, Hunt, Preheim, & Weinreich, 2006). On the contrary, complex traits that involve multiple functional genes tend to be conserved at a high rank in the phylogeny (Martiny et al., 2015). Altogether, these considerations highlight the limited functional resolution provided by the single‐gene (16S, ITS, and 18S) vision of microbial biodiversity, and the need for more functionality‐oriented, trait‐based approaches (Allison, 2012; Bodelier, 2011; Fierer et al., 2014; Green et al., 2008; Hillebrand & Matthiessen, 2009; Krause et al., 2014; Litchman, Edwards, & Klausmeier, 2015; Nemergut et al., 2013; Shade & Handelsman, 2012; Shade et al., 2012; Wallenstein & Hall, 2012).

### 2.3 Differences between micro‐ and macroorganisms in a functional context and limitations of current theoretical frameworks

There are some concepts that pertain to biodiversity and functional relationships in both macro‐ and microbial ecology. For example, the positive effect of biodiversity on ecosystem functioning is usually attributed to two nonexclusive mechanisms, the selection (or sampling) and the complementarity effects (Cardinale et al., 2006; Loreau, Mouquet, & Gonzalez, 2003; Loreau et al., 2001). To put it simply, selection effect reflects the influence of a single hyper competitive species on the overall community function, while complementarity effect depends on the presence of species with complementary traits and results from resource partitioning or facilitation among them. Both concepts relate directly to the fact that organisms' traits determine their impact on ecological process under study.

But, there are prominent differences between micro‐ and macroorganisms that prevent direct transfer of ecological theories and concepts. These include the small size of individual microbes that contribute to their greater sensitivity to environmental change, their faster metabolic, and growth rates, but also the colonial growth of microbes which is opposed to the unitary construction of most macroorganisms (Plante, 2017). Beside these general considerations, microbial functional ecology faces several major challenges that prevent the direct application of concepts and methods developed for macroorganisms.

In functional ecology of macroorganisms, the traits are often measured at the species level, a concept whose existence is highly debated for microbes (Gevers et al., 2005; McLaren & Callahan, 2018). The classic approach in microbial ecology was based on a similar unit, the isolated strain (i.e., the colony formed by a single cell), and the picture of the community was constructed using the taxonomic classification of the isolated strains. It is worth noting that the species is still used as the reference unit in the fields of pathogenic bacteriology and food microbiology. Nonetheless, modern molecular microbial ecologists are using a proxy, the operating taxonomic unit (OTU), that is defined by grouping sequences amplified from a single marker gene (e.g., 16S, 18S, ITS) using DNA extracted at the community level (Konopka, 2009; Schloss & Westcott, 2011). Recently, amplicon sequence variants (ASV) have been proposed as replacement of OTUs in microbial ecology, but ASV share similar limitations as OTUs in a functional context (Callahan, McMurdie, & Holmes, 2017; Glassman & Martiny, 2018). Hence, the unit assayed in molecular microbial ecology is the community, and its individual components are identified a posteriori.

The species or OTU unit is also problematic because it requires a library matching traits to genes or OTUs and it neglects intraspecific variability. Most environmentally important microbes have yet to be cultivated, and most functional traits can only be validated using culturable taxa. Consequently, limited physiological, physical, and metabolic information is available for assessing functional diversity of individual taxa (Schnoes, Brown, Dodevski, & Babbitt, 2009; Turaev & Rattei, 2016) and inference of function from taxonomy/phylogeny may only apply to specialized and well conserved functions, such as methanogenesis (Goberna & Verdú, 2016). The existence of HGT (Polz et al., 2006) and the poorly defined concept of prokaryotic species (Gevers et al., 2005) make such a linkage even more difficult. Based on the metabolic or physiological traits measured on culturable taxa, many of these traits differ from one taxon to another and for most functions there exists little‐to‐no taxonomic resolution (Louca et al., 2018; Martiny et al., 2015). The functional approach, especially when applied to microbes, addresses the problem of taxa‐traits associations by assessing the community as a multivariate and continuous distribution of traits. Doing so, one could characterize communities using the frequency of different trait values and forget about taxonomic diversity.

Another challenge is presented from the fundamental differences in the nature of the traits measured. Indeed, macroorganisms traits are often constitutive, that is, continuously expressed, and exist in the ecosystem as long as the organism is alive (e.g., the shape of a plant's leaf or the size of a fish's mouth). While this can also be the case in microbes, for instance in bacterial cells that possess pili or phytoplankton organisms with hard shells, the expression of microbial traits tends to be more directly related to their environment. Indeed, the link between genotype and phenotype is narrower in microbes than macroorganisms (Dutilh et al., 2013; Tamura & D'haeseleer, 2008). Hence, many microbial traits are genetically regulated (e.g., metabolic pathways, biofilm formation, and virulence) and their induction dependent on population size, cell activity, and environmental conditions.

Despite the above‐mentioned constraints, microbes likely represent the best system to apply functional approaches. On one hand, defining species is controversial if not impossible because of gene transfers and asexual reproduction, the diversity is astonishing and sampling constraints make it difficult to measure traits and functions. On the other hand, functional redundancy is widespread, the relative simplicity of microbial physiology facilitates the mapping of genes to functions and novel sequencing methods allow the documentation of many genes simultaneously. The functional approach may thus appear as a solution to reduce the complexity of microbial systems and better understand their functioning. It is worth noting that the field of microbial functional ecology is pretty new and it is not common to apply function diversity measures to characterize microbial communities.

## 3 DEFINING MICROBIAL FUNCTIONAL DIVERSITY

Here, we do not suggest a universal definition of microbial functional diversity as it appears that there is no such thing. Instead, we present synthesis of the literature suggesting that two visions of microbial functional diversity have emerged, which mostly differ in the ecological unit on which traits are measured. Some authors propose to measure traits at the taxa level while others suggest that measuring traits directly at the community level is more relevant. In the following sections, we will discuss the rationale between both approaches along with the required data and the insights that could be gained. By clarifying the debate about microbial functional diversity estimation, we should be able to go beyond simply “studying microbial functional diversity” and move toward a more precise understanding of the factors shaping functionality of microbial communities at various scales.

### 3.1 Functional units in microbial ecology

#### 3.1.1 Taxa‐centered approaches

In microbial ecology, the taxon‐centered approach has been termed “genome‐centric” or “organism‐based” (Alivisatos et al., 2015; Prosser, 2015; Turaev & Rattei, 2016), but it is conceptually similar to what is currently done in macrobial functional ecology and requires the same type of data, that is, “taxa‐traits” matrices. However, as highlighted in previous sections, such data are difficult to obtain for microbes using current methods. Developing such approaches would undoubtedly provide valuable insights into the functional structure of the microbial world. For instance, one could represent the functional niche of microbial taxa as proposed by Hutchinson (1957), as a multidimensional hypervolume in which each axis is a trait (Lennon, Aanderud, Lehmkuhl, & Schoolmaster, 2012). Doing so, we could better understand ecological strategies and processes underlying community patterns in an environmental context (Green et al., 2008; Nemergut et al., 2013), tackle questions related to the niche versus neutral theory debate, test the linkage between phylogeny and function (Prosser, 2015), determine which functions exhibit higher or lower functional redundancy (Yachi & Loreau, 1999), or identify functional trade‐offs or covariation between traits. Delineating the functional niche of taxa rather than the potential of the community will allow us to determine who is doing what in the community and thus to better identify vulnerable communities (i.e., communities in which some functions are realized by few and/or vulnerable taxa). Furthermore, taxon‐trait approaches would allow us to identify taxa with unique functional potential that may be irreplaceable within their ecosystem, that is, “keystone species.” Controlled experiments based on artificially assembled communities can enable defined taxon‐trait associations (Krause et al., 2014; Wallenstein & Hall, 2012) but such reductionist approaches are difficult to extend to the far more complicated large‐scale studies of natural communities (Fierer & Lennon, 2011; Fierer et al., 2014). Building frameworks for these assessments, expanding access to full microbial genomes and extracting ecological relevant traits from these will be essential to the future of microbial functional ecology. These advances may mark a paradigm shift when the field of microbial ecology transitions from the taxonomic/phylogenetic approach toward a classification based on environmental roles and functional performance (Krause et al., 2014).

#### 3.1.2 Community‐centered approaches

The most widely used approach in microbial functional ecology consists of comparing communities using directly their traits, which often reduce to the composition and abundance of particular genes. However, metagenomics produce data suitable for extending trait‐based analyses from the taxon to the community level, thus eluding the confounding effects of HGT present at lower levels of organization (Barberan, Fernandez‐Guerra, Bohannan, & Casamayor, 2012). Indeed, it seems that there is no level of taxonomic resolution that unambiguously translates into functional differentiation, notably because microbes are redundant in many of the function they carry (Louca et al., 2018). This is supported by the frequent observation of a decoupling between taxonomic and functional community composition (Cheaib, Boulch, Mercier, & Derome, 2018; Goldford et al., 2018; Louca, Parfrey, & Doebeli, 2016; Mouchet et al., 2012; Roth‐Schulze, Zozaya‐Valdés, Steinberg, & Thomas, 2016). Along this idea, an increasing number of studies proposed that the unit of microbial ecology should be the genes rather than individual taxa (Boon et al., 2014; Konopka, 2009; Louca et al., 2018; Miki, Yokokawa, & Matsui, 2013). Others advocated for a “microbial ecology without species,” asking whether the partition of a community into species/OTUs is an adequate description of the microbial world (Tikhonov, 2017). This is in line with the system biology idea that a community is more than the sum of its parts and exhibits emergent properties resulting from the attributes of an assemblage of organisms that live together in a contiguous environment and interact with each other (Boon et al., 2014; Fierer et al., 2014; Goldford et al., 2018; Konopka, 2009). This also agrees with the idea that microbial communities' adaptation to environmental conditions can be achieved through changes in the relative contribution of microbial populations to the total aggregated function of the community (Wallenstein & Hall, 2012) and is in accordance with the mass ratio hypothesis (Grime, 1998). As a result, community‐level traits should be easier to link with community‐level properties (Fierer et al., 2014) as it is done in plant functional ecology with the use of community‐aggregated traits (Lavorel & Grigulis, 2012). This approach appears more adapted for the study and comparison of natural communities at large scales. Thus, we propose that assessing microbial functional diversity using community‐level traits represents a step forward for characterizing the functioning of microbial systems and studying microbial functional biogeography (Bier et al., 2015; Violle, Reich, Pacala, Enquist, & Kattge, 2014).

### 3.2 Functional traits in microbial ecology

#### 3.2.1 Genotypic microbial traits

The most commonly used microbial traits in the post‐NGS era are genotypic traits. These encode the functional potential of microbes and mostly correspond to the presence of particular functional genes or pathways in the genome of microbial taxa or their abundance in the metagenome of a community (Goberna & Verdú, 2016).

In a taxa‐centered perspective, genome reconstruction using sequencing approaches is the most commonly acknowledged method to describe the functional potential of a taxon using genotypic traits. While this can be relatively straightforward for cultivated organisms, it can become quickly intractable in environmental samples containing an enormous number of organisms. The first automated softwares for genome reconstruction in environmental samples have been developed (e.g., MAPLE: Takami, 2019; groopM: Imelfort et al., 2014; CONCOCT: Alneberg et al., 2014), and what seemed impossible not so long ago appears more and more feasible. For instance, Anantharaman et al. (2016) reconstructed more than a thousand almost‐complete (>93%) genomes, which represented a third of all microorganisms present in low biomass samples from an aquifer. Similarly, Takami (2019) reconstructed the genome of an uncultured archaea from a metagenomic library and inferred its physiological potential, expressed as the level of completion of KEGG modules and pathways. The completion level of functional pathways identified from metagenome may be more relevant than single functional genes and can easily be used as a functional trait. Beside the genome content in terms of genes, some authors have proposed to functionally characterize microorganisms using genotypic traits such as GC content, number of genes per genome, effective genome size, or 16S rRNA gene copy number (Barberan et al., 2012; Fierer et al., 2014; Goberna & Verdú, 2016). These “genome‐centric” approaches are highly promising and constitute an important prerequisite toward a systems‐level understanding of microbial communities (Turaev & Rattei, 2016), but the methods are still novel and their democratization to a wide range of users will likely take some time. Further, a worth noting drawback of genome reconstruction approach is that most metagenome‐assembled genomes are mosaics and not necessarily accurately portray a single species.

In a community‐centered perspective, a community can be defined as a functional library composed of a collection of genes that may be selected by a given set of environmental conditions (Boon et al., 2014; Burke et al., 2011; Miki et al., 2013; Wallenstein & Hall, 2012). There are two main sets of approaches to characterize the metagenomic content of a community. A first set of approaches infers the functional contents of a community using its taxonomic composition (e.g., PiCRUST: Langille et al., 2013; Tax4Fun: Aßhauer et al., 2015; Vikodak: Nagpal, Haque, & Mande, 2016). In environments with many reference genomes, the accuracy of functional inferences can be comparable to shotgun sequencing (Turaev & Rattei, 2016), but their accuracy in complex and poorly described systems is debatable (Iwai et al., 2016; Xu, Malmer, Langille, Way, & Knight, 2014). Additionally, these methods were designed to predict vertically inherited functions and are thus more powerful for functions strongly associated with the evolution of taxonomic lineages. A second set of approaches is based on shotgun sequencing of metagenome or metatranscriptome and subsequent functional categorization of sequences to functional genes or pathways (Alneberg et al., 2014; Carvalhais, Dennis, Tyson, & Schenk, 2012). Once the metagenomic content of a community has been determined, several genotypic traits may be estimated such as the GC content and its variation, effective genome size, or metagenome functional content (Barberan et al., 2012). Others proposed to use shotgun metagenomic sequencing to estimate community‐aggregated traits (Raguideau, Plancade, Pons, Leclerc, & Laroche, 2016) or to determine the molecular pathways contained by communities, their level of completion, and the diversity of organisms participating in these pathways (Takami, 2019). Another approach is to consider functional genes as community traits and to use the diversity of gene variants as trait value, as it reflects the diversity of organisms carrying the function associated with the gene. This approach has been mostly reduced to the total abundance of genes, estimated as the number of sequences in the community (Burke et al., 2011; Souza et al., 2015), the signal intensity on a functional gene array (Bai et al., 2013; Bayer et al., 2014), or quantification using qPCR (Philippot et al., 2013; Powell, Welsh, Hallin, & Allison, 2015). A finer characterization of gene variants diversity is possible by considering their richness (Huang et al., 2014) or evenness (Powell et al., 2015) instead of their sheer abundance. For instance, denitrification rates were more strongly linked to the evenness of *nir* genes variants abundance distribution than to their richness or the total number of *nir* gene copies (Powell et al., 2015). Further, it was proposed that the ecosystem processes most sensitive to changes will be those narrowly distributed among phylogenies (Treseder et al., 2012), as distantly related organisms are more likely to be adapted to different environmental conditions than closely related ones. Indeed, it was shown that, despite the fact that HGT can spread functions across taxonomic and phylogenetic barriers, dissimilarity among organisms supporting a function can promote its productivity and stability (Carrara et al., 2015; Salles, Poly, Schmid, & Le Roux, 2009). We foresee that these types of approaches will provide new insights by allowing the estimation and comparison of functional diversity at the community level.

For genotypic traits to become the standard for microbial functional ecology, this will require a validation of their actual linkage with phenotypic traits and associated ecological strategies. Toward this goal, we provide a list of more than 400 genotypic functional traits covering microbial functions related to biogeochemistry, ecology, environmental sciences, and human health (Table 1 and Table S2). The characterization and classification of functional genes presented in these tables were realized using information available in databases such as NCBI, UniProt, or EXpasy and also were based on extensive literature reviews.

**Table 1:** Microbial genotypic functional traits important to biogeochemical cycling, organic contaminant degradation, stress responses, antibiotic resistance, and virulence

Ecosystem process	Functional process	Microbial functional trait	Organism's performance	Organism's fitness	Community/ecosystem level functions
Carbon cycling	CO_2_ fixation	Ribulose bisphosphate carboxylase (rbcL)	CO_2_ fixation	Growth and reproduction	Carbon fixation; calvin cycle
		Mg‐protoporphyrin IX chelatase (chlI)	CO_2_ fixation	Growth and reproduction	Carbon fixation, chlorophyll production
		Tetrahydrofolate formylase (fhs)	Transform formate and tetrahydrofolate to 10‐formyltetrahydrofolate	Growth and reproduction	Acetogenesis, carbon fixation
	Methanogenesis	Methyl coenzyme M reductase (mcrA)	Transform of methyl‐CoM to methane	Growth and reproduction	Methanogenesis
	Methane oxidation	Particulate methane monooxygenase (pmoA)	Oxidize methane to methanol	Growth and reproduction	Methane oxidation
	Carbon degradation	Endoglucanase	Cellulose degradation	Growth and reproduction	Biomass degradation, cellulose
		Chitinase	Chitin degradation	Growth and reproduction	Biomass degradation, chitin
		Xylanase (xynA/xynB)	Hydrolysis of 1‐,4‐beta‐D‐xylosidic linkages in xylans	Growth and reproduction	Biomass degradation, hemicellulose
		Manganese peroxidase (mnp)	Lignin degradation	Growth and reproduction	Biomass degradation, lignin
		Pectin lyase (pelA)	Cleavage of (1‐>4)‐alpha‐D‐galacturonan methyl ester	Growth and reproduction	Biomass degradation, pectin
		Alpha‐amylase (amyA)	Endohydrolysis of 1,4‐alpha‐D‐glucosidic linkages	Growth and reproduction	Biomass degradation, starch
		Protease	Protein degradation	Growth and reproduction	Biomass degradation, protein
Nitrogen cycling	N mineralization	Urease (UreC)	Hydrolyzing urea into ammonia	Growth and reproduction	N mineralization
		Glutamate dehydrogenase	Fixation of ammonia into organic matter or oxidation of glutamate	Growth and reproduction	N mineralization
	Anammox	Hydrazine oxidoreductase (hzo)	Transformation of N_2_H_4_ to N_2_	Growth and reproduction	Anammox
	N reduction	Nitrate reductase, assimilatory (nasA/narB)	Reduction of NO_3_ to NO_2_	Growth and reproduction	Assimilatory N reduction
		Nitrate reductase, respiratory (napA)	Reductions of NO_3_ to NO_2_	Growth and reproduction	Dissimilatory N reduction
	Nitrification	Ammonia monooxygenase (amoA)	Oxidation of ${\text{NH}}_{4}^{+}$ to NH_2_OH	Growth and reproduction	Nitrification
		Hydroxylamine oxidoreductase (hao)	Oxidation of NH_2_OH to NO_2_	Growth and reproduction	Nitrification
	Denitrification	Nitrate reductase, respiratory (narG)	Reduction of NO_3_ to NO_2_	Growth and reproduction	Denitrification
		Nitrite reductase (nirS/nirK)	Reduction of NO_2_ to NO	Growth and reproduction	Denitrification
		Nitrous oxide reductase (nosZ)	Reduction of N_2_O to N_2_	Growth and reproduction	Denitrification
	N Fixation	Nitrogenase (nifH)	Fixation of N	Improved growth, survival, and reproduction	N fixation
Phosphorus utilization	Organic	Exopolyphosphatase (ppx)	polyP degradation	Nutrient storage; Growth and reproduction	Phosphorus storage
	Inorganic	(ptxD)	Phosphorus oxidation	Growth and reproduction	Phosphorus acquisition
Sulfur utilization	Reduction	Adenylylsulfate reductase (aprA)	Production of adenylyl sulfate from sulfite	Growth and reproduction	Adenylylsulfate reductase
		Dissimilatory sulfite reductase (dsrA)	Oxidation of H_2_S to SO_3_	Growth and reproduction	Sulfur reduction
	Oxidation	Sulfur oxidation protein SoxY	Carrier protein for thiosulfate oxidation	Growth and reproduction	Sulfur oxidation
	Assimilation	Dimethyl sulfoniopropionate demethylase (dmdA)	Oxidation of DMSP	Growth and reproduction	Sulfur acquisition (marine)
Metal homeostasis	Arsenic	Arsenate reductase (arsC)	Reduction of arsenate to arsenite	Survival	Detoxification
	Mercury	Mercuric reductase (merA)	Reduction of Hg^2+^ to Hg^0^	Survival	Detoxification
	Multiple metals	Heavy metal efflux pump CzcA	Resistance to Cadmium, cobalt, zinc	Survival	Transport pump
	Multiple metals	Metallothionein (smtA)	Sequestration of metal ions	Survival	Sequestration
	Iron	Bacterioferritin (bfr)	Binding of iron ions	Survival, growth, and reproduction	Storage
Organic contaminant remediation	Aromatic carboxylic acid	Phthalate 4,5‐dioxygenase (phtA)	Oxidation of phthalate	Survival, growth, and reproduction	Detoxification/energy generation
	BTEX and related aromatics	Toluene 2‐monooxygenase (tomA3)	Oxidation of toluene	Survival, growth, and reproduction	Detoxification/energy generation
	Chlorinated aromatics	2,4‐Dichlorophenoxyacetate alpha‐ketoglutarate dioxygenase (tfdA)	Oxidation of herbicide	Growth and reproduction	Detoxification/energy generation
	Heterocyclic aromatics	Dioxin dioxygenase/dibenzofuran dioxygenase (dxnA)	Oxidation of Dibenzo‐p‐dioxin and Dibenzofuran	Survival, growth, and reproduction	Detoxification/energy generation
	Other aromatics	Catechol 1,2‐dioxygenase	Conversion of catechols to cis, cis‐muconates	Survival, growth, and reproduction	Detoxification/energy generation
	Polycyclic aromatics	PAH‐inducible cytochrome P450 monooxygenase (p450aro)	Oxidation of PAHs	Survival, growth, and reproduction	Detoxification/energy generation
	Chlorinated solvents	Reductive dehalogenase (rd)	Reductive dehalogenation	Survival, growth, and reproduction	Detoxification/energy generation
	Herbicides related compounds	Atrazine chlorohydrolase (atzA)	Degradation/utilization of triazine herbicides as a nitrogen source	Survival, growth, and reproduction	Detoxification/nitrogen utilization
	Other Hydrocarbons	Alkane 1‐monooxygenase (alkB)	Oxidation of alkanes	Survival, growth, and reproduction	Detoxification/energy generation
	Pesticides related compounds	Haloalkane dehalogenase	Dehalogenation of synthetically produced haloalkanes	Survival, growth, and reproduction	Detoxification/energy generation
Antibiotic resistance		Beta‐lactamase	Degradation of beta‐lactam containing compounds	Survival, growth, and reproduction	Detoxification/energy generation
		Glutathione transferase fosA	Enzymatic alteration of fosfomycin	Survival, growth, and reproduction	Detoxification
		Quinolone resistance protein	Binds to enzymes targeted by quinolone antibiotics	Survival, growth, and reproduction	Resistance and resilience to quinolone antibiotics
Stress responses	Acid	Acid shock protein	Survival under acidic condition	Survival, growth, and reproduction	Resistance and resilience to environmental changes/perturbations
	Alkaline	Alkaline shock protein	Survival under alkaline condition	Survival, growth, and reproduction	Resistance and resilience to environmental changes/perturbations
	Temperature	Cold shock protein A	Survival of sudden temperature drops (cold shock)	Survival, growth, and reproduction	Resistance and resilience to environmental changes/perturbations
	Desiccation	Trehalose synthase	Production of trehalose to help maintain cytoplasm integrity (drought tolerance)	Survival, growth, and reproduction	Resistance and resilience to environmental changes/perturbations
	Envelope	Phage shock protein A (pspA)	Helps maintain proton motive force	Survival, growth, and reproduction	Resistance and resilience to environmental changes/perturbations
	Nitrogen limitation	Glutamine synthetase (glnA)	Production of glutamine from glutamate	Survival, growth, and reproduction	Resistance and resilience to environmental changes/perturbations
	Oxidative stress	Superoxide dismutase	Conversion of superoxide to hydrogen peroxide	Survival, growth, and reproduction	Resistance and resilience to environmental changes/perturbations
	Oxygen limitation	Transcriptional regulator fnr	Regulator that becomes active under oxygen limitation	Survival, growth, and reproduction	Resistance and resilience to environmental changes/perturbations
	Phosphate limitation	Phosphate ABC transporter, ATP‐binding protein (pstB)	Uptake of inorganic phosphate	Survival, growth, and reproduction	Resistance and resilience to environmental changes/perturbations
	Protein stress	Serine endoprotease	Protein quality control under environmental stresses	Survival, growth, and reproduction	Resistance and resilience to environmental changes/perturbations
	Stringent response	GTPase	Keeps intracellular ppGpp concentrations low	Survival, growth, and reproduction	Resistance and resilience to environmental changes/perturbations
Virulence	Toxin	Cytolethal distending toxin B (cdtB)	DNase; host cell death and release of nutrients	Survival, growth, and reproduction	Release of nutrients
	Infection	Circumsporozoite protein	Colonization/infection of host cells	Survival, growth, and reproduction	Infection of host organisms

Note

A more comprehensive, but not exhaustive, table is presented in Table S2. Characterization and classification of functional genes presented in these tables were realized using information available in databases such as NCBI, UniProt, or EXpasy but also based on extensive literature reviews.

#### 3.2.2 Phenotypic microbial traits

The expression of genotypic traits at the taxon or community level results in phenotypic traits, that are conceptually more similar to the traits measured for macroorganisms. These traits are expected to be more directly related to ecosystem processes than genotypic ones, but this will depend on the process considered and our ability to estimate a phenotypic trait related to the process of interest.

In a taxa‐centered perspective, phenotypic traits include organisms' characteristics (e.g., cell dimensions, shape, motility, spore formation, growth rate, stoichiometry), environmental preferences (e.g., oxygen requirement, optimal pH, temperature, and salinity tolerance) and metabolic capabilities (e.g., production of certain enzymes Barberán, Caceres Velazquez, Jones, & Fierer, 2017; Goberna & Verdú, 2016). Phenotypic traits can sometimes be inferred from genotypic ones (Barberán et al., 2017) but they are mostly estimated using cultured organisms and generally measured in controlled and/or favorable conditions. Hence, there is little certainty that a phenotype associated with a taxon in the laboratory will also be observed in situ but this is a common weakness of cultivation‐based and cultivation‐free approaches. The scarcity of taxon‐phenotypic traits data is even more pronounced than for genotypic traits, and this is notably due to lack of culturable representatives for most microbial groups (Aslam, Yasir, Khaliq, Matsui, & Chung, 2010; Pham & Kim, 2012). However, Barberán et al. (2017) collected phenotypic and environmental tolerance traits from articles published in the International Journal of Systematic and Evolutionary Microbiology (IJSEM), yielding the characterization of more than 5,000 bacterial strains. This database represents the typical type of taxon‐trait data required to bridge the gap between functional ecology of micro‐ and macroorganisms through the use of common methods previously limited to macrobial organisms.

In a community‐centered perspective, phenotypic traits can correspond to community‐aggregated traits (CATs, Fierer et al., 2014), but often correspond to estimates of community functioning sensus studies relating biodiversity and ecosystem functioning (BEF). For instance, this includes substrate use profiles (e.g., Biolog), production of gases, degradation of compounds, or temporal stability of biological process (e.g., biomass production, rate of compound degradation). These community‐level phenotypic traits are much more difficult to predict than their taxon‐level counterparts, notably as they arise from the interaction of many organisms. Novel methods provide the opportunity to identify functional groups within communities based on their activity or physiological states. For instance, stable isotope probing (SIP) allows the identification of groups of organisms that assimilate a particular substrate that was isotopically labeled beforehand. Doing so, it is possible to identify groups of organisms that behave similarly regarding particular compounds, or in other term to define a microbial functional group regarding a substrate. In soil systems, SIP can be combined with high‐resolution secondary ion mass spectrometry (NanoSIMS) or Raman microspectroscopy to get insights into the in situ function of microorganisms (Eichorst et al., 2015). In aquatic and soil environments, SIP can be coupled with flow cytometry (FCM) and cell sorting to identify microbial functional groups within a water sample (Couradeau et al., 2019; Pjevac et al., 2019). In addition, FCM generates huge quantities of data that contains many parameters reflecting the physiological states of the cells. These multidimensional data can then be used as traits to identify and quantify functional groups of cells within the community (Props, Monsieurs, Mysara, Clement, & Boon, 2016).

#### 3.2.3 Other considerations regarding microbial functional traits

Beside the genotype/phenotype dichotomy, other considerations exist regarding the concept of functional trait. The first one concerns the distinction between effect and response traits (Allison & Martiny, 2008; Zwart, Solomon, & Jones, 2015). Effect traits relate to the concept of ecological niche (Elton, 1927), that is, they define organisms' ecological role by governing their ability to realize and influence ecological processes (Allison & Martiny, 2008). Response traits relate to the concept of environmental niche (Grinnell, 1917), that is, they define organisms' ability to respond to and withstand changes of environmental conditions (Allison & Martiny, 2008). The distinction between effect and response traits is considered as critical for biodiversity and ecosystem functioning studies (Naeem & Wright, 2003), but their distinction appears highly complex for microbes. The choice of traits will depend on the question of interest, and whether one is interested in the realization of a particular ecological process involving effect traits or in the stability of a process across time, space, or environmental gradients, which depends on the ability to respond to environmental changes (Jurburg & Salles, 2015; Mori, Furukawa & Sasaki, 2013; Naeem & Wright, 2003). A second important distinction separates fundamental and realized ecological niches (Hutchinson, 1957) as it allows differentiating the intrinsic taxon/community attributes (fundamental) from the contingent properties dependent on abiotic and biotic environments (realized; Devictor et al., 2010; Martiny et al., 2015). In the context of genotypic traits, fundamental and realized functionality of microbes can be assessed using DNA or RNA‐based approaches, respectively. For instance, the use of (meta)transcriptomics provides information about which genotypic traits are actively expressed, allowing the identification of the processes realized by the studied units or the types of environmental stresses they are dealing with Moran et al. (2013). Ideally, fundamental and realized niches should be assessed simultaneously to determine whether observed differences in realized functions arise from different environmental conditions acting on a similar functional potential or from different potential between communities (Louca et al., 2018). For a recent review on the topic of ecological niches of microbes, the reader is invited to read the brilliant piece on microdiversity by Larkin and Martiny (2017).

## 4 EXAMPLE OF APPLICATIONS

### 4.1 Estimating microbial functional diversity using a taxa‐traits approach

**Figure 1:**
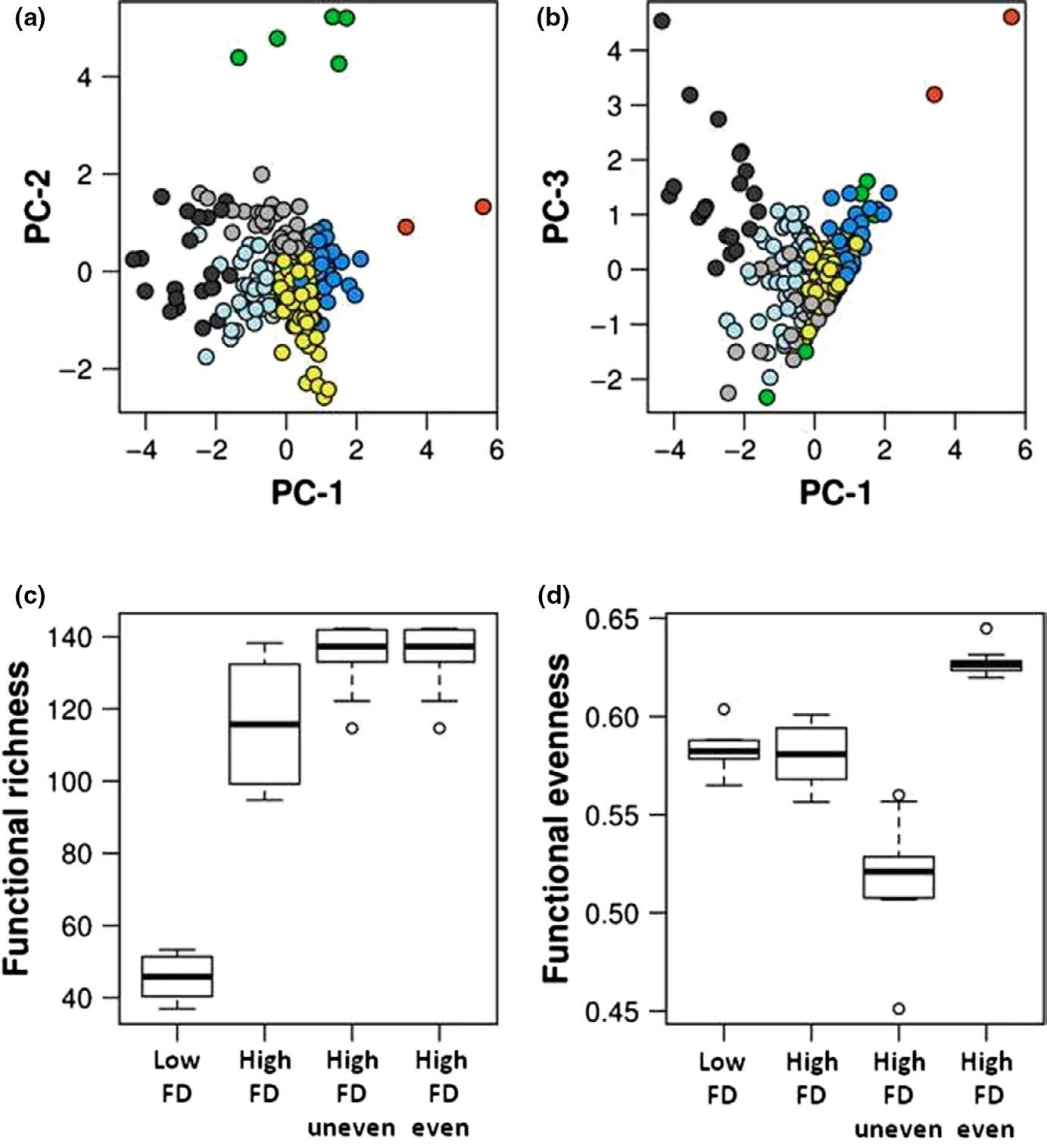
Estimation of microbial functional diversity using a taxa‐traits approach. (a) and (b) represent the position of 248 soil bacterial species in the functional space defined by three continuous response traits; growth optima in salinity, pH, and temperature. Species were clustered into seven functional groups (dots with different colors) exhibiting different trait values and defined using Kmeans classification. (c) and (d) represent two facets of functional diversity estimated on four sets of communities with contrasted functional characteristics (see main text for details): functional richness (c) and functional evenness (d)

Here, we present an example of functional diversity estimation following a taxa‐traits approach and using tools developed for macroorganisms. To this end, we used the database released by Barberán et al. (2017), which contains the phenotypic and environmental tolerance traits for more than 5,000 bacterial strains. From this database, we selected 298 species from the soil habitat characterized by three continuous response traits corresponding to the species' optimal growth conditions in terms of salinity, pH, and temperature. These traits were used to define functional groups of species, that is, groups of species with similar traits. Groups colored in green and orange were the most functionally dissimilar, whereas black was also functionally distinct but to a lesser extent (Figure 1a,b).

Then, we generated four sets of communities (*n* = 10) exhibiting contrasted functional characteristics. The first two sets were composed of 210 ± 5 species spanning a small (*lowFD*, no species from the green and orange functional groups) and high (*highFD*) range of trait values, respectively. The third and fourth sets were composed of 264 ± 5 species with similar trait values but different distribution of species across trait values. One set of communities was dominated by species with intermediate trait values (*highFD*‐*even*) while the other was dominated by species with extreme trait values (*highFD*‐*uneven*, dominance by species from the orange and green functional groups). We used the R package *FD* to estimate two different facets of functional diversity, richness, and evenness (Laliberté, Legendre, & Shipley, 2015). Functional richness (Figure 1c) is the amount of functional space occupied by the organisms composing the community and represents the range of traits observed in the community (Villéger et al., 2008). As expected, the two sets of communities with more species have a higher functional richness (*highFD*‐*even* and *highFD*‐*uneven*). In other terms, the range of environmental conditions in which the species composing the community are capable of growth is larger. Additionally, the two sets with a lower species richness have a lower functional richness, but more importantly they differ greatly in their functional richness, with the communities composed of species with a narrower range of trait values (*lowFD*) having a lower functional richness. Functional evenness (Figure 1d) is the degree of regularity in the distribution of species abundance in the functional space occupied by the community. As expected, communities with abundance unevenly distributed across the trait values (*highFD‐uneven*) exhibited lower functional evenness than communities with more even distribution (*highFD‐even*). Further, the first two sets of communities exhibited similar functional evenness despite exhibiting different functional richness.

In this case study, we have shown that taxa‐traits approaches allow to cluster species according to their biological characteristics. Then, we can scale at the community level and estimate complementary facets of functional diversity to uncover differences between communities that relate to their ecological functioning.

### 4.2 Community‐level approach of microbial functional diversity

In the following sections, we provide two examples of community‐centered analyses of microbial functional diversity using ad hoc procedures. These two case studies are based on data from previous studies (Wu et al., 2017; Yue et al., 2015).

#### 4.2.1 Using gene variants diversity as trait increases the correlation between community traits and ecological processes

**Figure 2:**
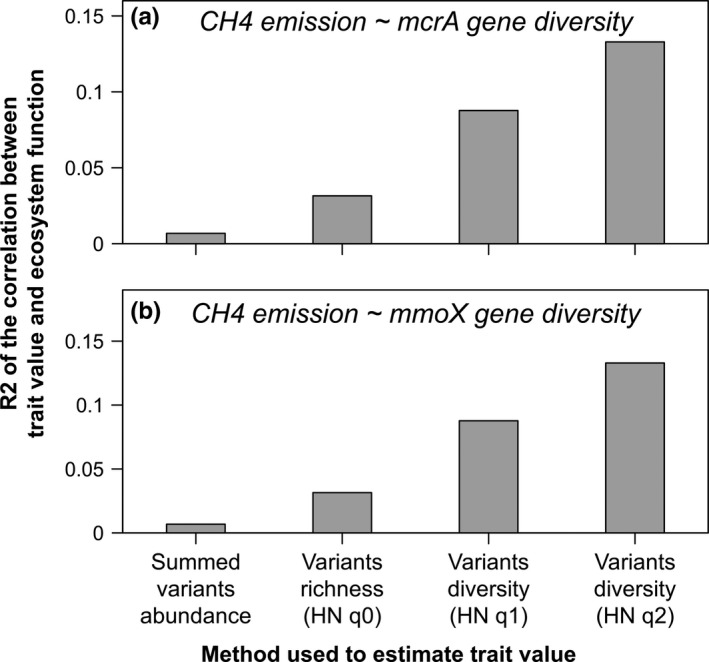
Gene variants diversity increases the correlation between community traits and ecological processes. HN q0, q1, and q2 correspond to Hill Number‐based gene variants diversity estimated with order (q) 0, 1 and 2, respectively

In this case study, we show how considering the diversity of functional genes variants at the community level can increase the correlation between estimated trait values and measured ecological processes (Figure 2a,b). We used data from previous studies (Wu et al., 2017; Yue et al., 2015) and analyzed the relationship between CH_4_ emission in natural grassland and the diversity of *mcrA* and *mmoX* genes estimated at the community level using the methodology described hereafter. The *mcrA* gene encodes the alpha subunit of methyl coenzyme M reductase, involved in the final step of methanogenesis (Ma, Conrad, & Lu, 2012) while the *mmoX* gene encodes a methane monooxygenase, involved in the first step of methane oxidation by methanotrophs (Murreil, Gilbert, & McDonald, 2000).

The starting data correspond to a table containing the functional genes detected across communities as typically obtained using metagenome sequencing or functional gene arrays. Each functional gene is represented by different variants that are associated with a presence–absence or abundance (number of sequences, hybridization intensity) within each community. Here, we used Geochip 4.0 data to estimate trait values with four different methods: (1) total gene abundance estimated as the sum of gene signal intensity; (2–4) diversity of gene variants estimated using Hill numbers (Chao, Chiu, & Jost, 2014). Hill numbers are a parametric family of diversity indexes differing among themselves only by the parameter *q* (called the order) that determines the sensitivity of the index to species relative abundances. Hill numbers generalize classic diversity indexes (Shannon and Simpson) and offer several advantages compared with these (i.e., Hill numbers obey the replication principle and are expressed in intuitive units of effective numbers of functional gene variants).

Then, we correlated the estimated trait values (i.e., diversity of functional genes variants) with community‐level functions (CH_4_ fluxes) and compared the explanatory power of the various methods. As shown in Figure 2a,b, considering the diversity of *mcrA* and *mmoX* genes variants can increase the explanatory power of traits compared with a simple aggregation of variants abundances.

#### 4.2.2 Characterizing the distribution of microbial functional traits within soil communities

**Figure 3:**
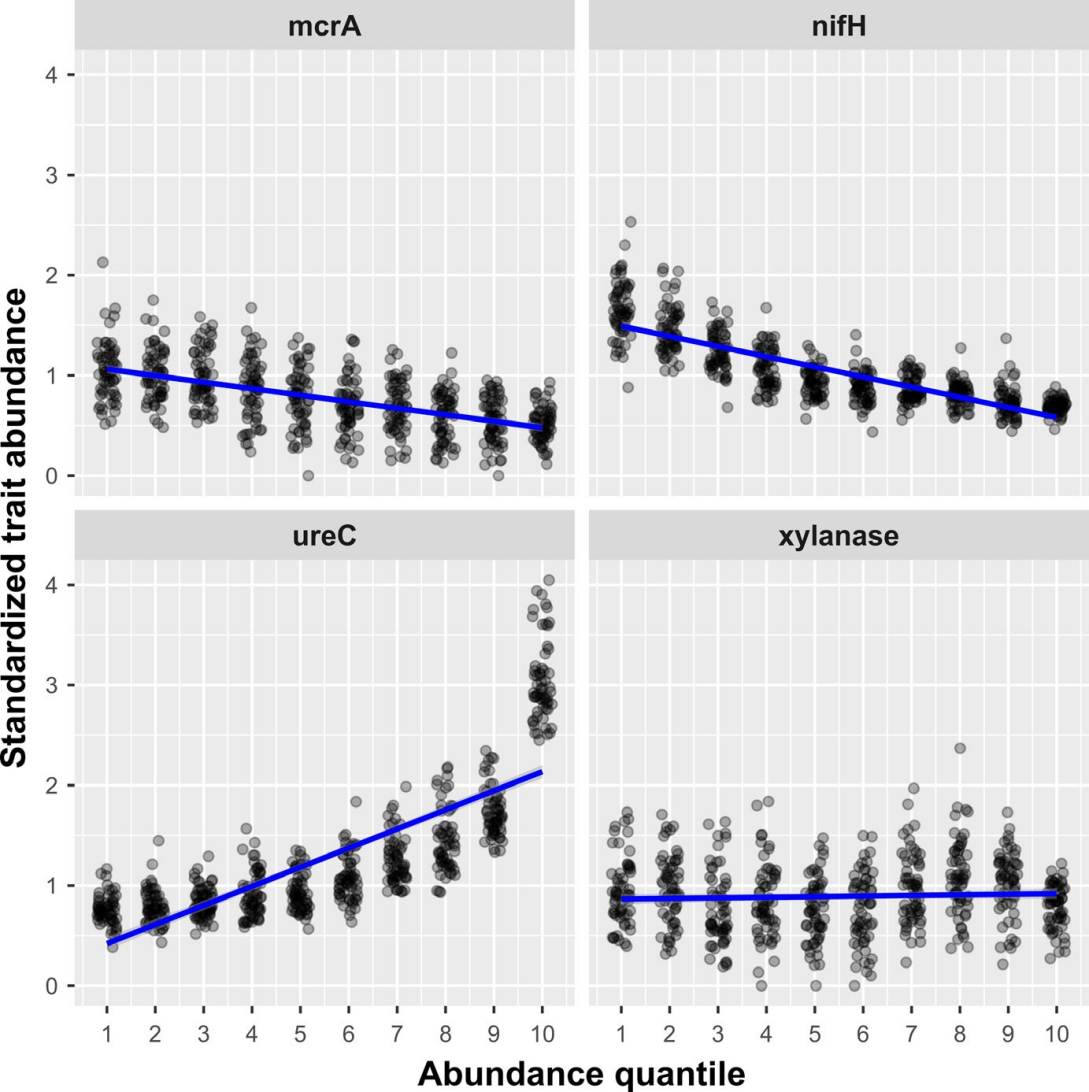
Distribution of functional traits within soil communities

In this last case study, we demonstrate that gene‐centered approaches can be used to characterize the distribution of functional traits within soil microbial communities and more particularly along a gradient from rarity to prevalence. Here, we used the same data as for the previous case study (Wu et al., 2017; Yue et al., 2015). The dataset consisted of 60 soil communities characterized using functional gene arrays (FGA, composed of 39,681 variants representing 194 traits). We determined the rank‐abundance distribution of all the variants within communities. We used the signal intensities of variants to order them along a rare to prevalent spectrum that corresponded to ten abundance quantiles. The abundance of traits carried by variants from each quantiles can then be estimated to identify traits that are over/underrepresented along this spectrum (Figure 3). For instance, both the *nifH* and the *mcrA* genes are underrepresented in the pool of abundant variants. In addition, *mcrA* gene is overrepresented in the pool of rare variants. On the contrary, the *ureC* gene is increasingly more abundant in pools of variants with increasing abundance. Finally, the *xylanase* gene does not show any trend and is thus present in similar abundance along the rarity to prevalence gradient.

In this case study, we showed that functional traits are distributed differently within microbial communities, with some traits being overrepresented in the pool of abundant variants while others being more present in the pool of rare variants. Highlighting such patterns has the potential to provide new insights into the functional organization of microbial systems.

## 5 CONCLUSIONS

The theories and mechanisms describing microbial ecology and biogeography can and should be improved. As highlighted herein, microbial ecology has unique sets of challenges from those of the macrobial world. But, the capacity to perform community‐wide molecular analyses is far less limited in microbial communities. Hence, with a molecular era, it is important to identify what data will be the most relevant. In most studies, the questions of interest correspond to function, so we propose that functional diversity receive attention. Microbial functional ecology is at a key moment of its structuring, novel tools are being continuously developed (Langille et al., 2013; Takami, 2019), data are made available (Barberán et al., 2017; Huse et al., 2014; Meyer et al.., 2008), and pioneers are paving the way for a rapidly advancing field (Fierer et al., 2014; Louca et al., 2018; Raguideau et al., 2016). In order to fulfill its promises, the field of microbial functional ecology will require solid foundations and we hope the synthesis and perspectives presented here will stimulate the thought and discussions toward this goal.

## CONFLICT OF INTEREST

None declared.

## AUTHORS' CONTRIBUTION

AE, JZ, and MF initiated the research. AE and LH wrote the first draft manuscript. All the authors participated in the manuscript edition.

## Data Availability

The data supporting the results presented in this article are available on the Dryad platform under the https://doi.org/10.5061/dryad.m3r1r2t.
