# Supplementary material for: Microbial functional diversity: From concepts to applications
Source: Ecol Evol. 2019 Oct 2;9(20):12000–16. doi: 10.1002/ece3.5670 (PMC6822047; doi:10.1002/ece3.5670)
Supplement: Supplementary file 3 [file ECE3-9-12000-s003.docx]

**Table S1. Definitions related to the concept of functional diversity observed in the literature**

Characterization and classification of functional genes presented in these tables was realized using information available in databases such as NCBI, UniProt or EXpasy but also based on extensive literature reviews.

**Table S2. List of putative microbial genotypic functional traits**

Characterization and classification of functional genes presented in these tables was realized using information available in databases such as NCBI, UniProt or EXpasy but also based on extensive literature reviews.
